# Heavy metal detection in mulberry leaves: Laser-induced breakdown spectroscopy data

**DOI:** 10.1016/j.dib.2020.106483

**Published:** 2020-11-02

**Authors:** Liang Yang, Liuwei Meng, Huaqi Gao, Jingyu Wang, Can Zhao, Meimei Guo, Yong He, Lingxia Huang

**Affiliations:** a College of Animal Sciences, Zhejiang University, Hangzhou 310058, PR China; b Research and Development Department, Hangzhou Goodhere Biotechnology Co., Ltd., Hangzhou 311100, PR China; c College of Biosystems Engineering and Food Science, Zhejiang University, Hangzhou 310058, PR China

**Keywords:** Heavy metal, Copper, Chromium, Mulberry leaf, Laser-induced breakdown spectroscopy

## Abstract

Five copper or chromium stress levels were carried out on mulberry leaf, and 20 samples were collected for each metal stress level. A total of 100 samples (copper or chromium) were processed into uniform pressed pellet. The mulberry leaf pellet was placed on a sample platform of laser-induced breakdown spectroscopy (LIBS) system. A laser was used to ablate the sample pellet and generate the emission lines, the intensity and delay time of laser ablation were 80 mJ and 4 μs respectively. To reduce the acquisition errors, 16 different positions of each sample were ablated for 5 accumulation. Then, 80 spectra were collected per sample and the average of them was considered as the sample spectrum for subsequent analysis. Finally, a total of 200 spectra of copper and chromium in mulberry leaves with a wavelength range of 219–877 nm were obtained for calibration analysis [1].

## Specifications Table

**Table:** 

Subject	Spectroscopy
Specific subject area	Laser-induced breakdown spectroscopy: Analysis of heavy metal in food using atomic Spectrometry
Type of data	Excel file (numerical matrix)
How data were acquired	Instruments: Laser-induced breakdown spectroscopy Make and model and of the instruments used: Q-switched Nd:YAG pulsed laser (Vilte-200, Beamtech Optronics Co. Ltd., Beijing, China), intensified charge coupled device (ICCD) detector (iStar DH340T-18F-03, Andor Technology, Belfast, UK), echelle spectrometer (Mechelle 5000, Andor Technology, Belfast, UK), digital pulse delay generator (DG645, Stanford Research Systems, USA), three-dimensional (X–Y–Z) motion platform (Zolix Instruments Co. Ltd., Beijing) [2]
Data format	Raw
Parameters for data collection	Sample: 0.3 g, laser intensity: 80 mJ, delay time: 4 μs, collection repetition: 16 positions and 5 accumulation [3]
Description of data collection	Samples were dried at 80 °C for 3 h and then ground. Then, 0.3 g sample powder was weighed and pressed with 600 MPa for 2 min to generate a press pellet. Sample pellet was placed on motion platform and ablated by laser to generate emission line which was collected by the light collector.
Data source location	Institution: Zhejiang University City/Town/Region: Hangzhou Country: P. R. China
Data accessibility	Repository name: Mendeley Data Data identification number: NA Direct URL to data:https://data.mendeley.com/datasets/7k86d5z37f/draft?a=726dc94f-3a27–4e46-bfdb-9b26e4bad54e
Related research article	Yang L., Meng L.W., Gao H.Q., Wang J.Y. Zhao C., Guo M.M., He Y., Huang L.X., Building a stable and accurate model for heavy metal detection in mulberry leaves based on a proposed analysis framework and laser-induced breakdown spectroscopy, Food Chemistry. doi: 10.1016/j.foodchem.2020.127886[1].

## Value of the Data

• These data provide the response laws between laser-induced breakdown spectra and mulberry contaminated by heavy metal.
• Researchers in the fields of food science, environment monitoring, rapid detection and agriculture can be benefit from these data [4], [5], [6].
• These data might be used for study of response mechanism between heavy metal and LIBS, further to carried out development of rapid detection instrument and model for metal monitoring [7].

## 1 Data Description

The data is provided as Excel file. There are two matrices (22,015 × 100), which are spectral data of heavy metal copper and chromium. The matrix is 22,015 × 100, which means 22,015 rows and 100 columns. Each column represents the full-band spectrum of one sample. And each row represents one waveband of all 100 samples.

## 2 Experimental Design, Materials and Methods

Uniform Dashi mulberry leaves without diseases or other defects were harvested from a mulberry garden. The mulberry leaves were washed with deionized water and dried at room temperature. Then A total of 200 leaves were selected to carried out heavy metal contamination. Analytical reagents of K_2_CrO_4_ and CuSo_4_ were dissolved in deionized water to prepared the contamination solutions. Five contamination levels of Cu^2+^ (0, 500, 1000, 2000, and 4000 mg L^−1^) or Cr^6+^ (0, 100, 500, 800, and 1000 mg L^−1^) were designed to obtain a broad range of values for model calibration. Mulberry leaves were immersed in the solution for 36 h. Among them, each concentration level contained 20 leaf samples. The contaminated leaves were then rinsed by deionized water to remove the metal ions from the surface and dried at 80 °C for 3 h in an oven. Before the spectra acquirement, all the samples were processed into uniform pressed pellet (10 mm width, 10 mm depth, and 2 mm height) under the condition of 600 MPa pressure and 2 min. During the spectra collecting, sample pellet was placed on motion platform and ablated by laser (laser intensity: 80 mJ, delay time: 4 μs) to generate emission line which was collected by the light collector. Sixteen different position were chosed for 5 accumulation of laser ablation. The acquired spectral data was then analyzed using MATLAB 2015b software (The Math Works, Natick, USA).

## Ethics Statement

This article does not contain any studies with human participants or animals performed by the authors.

## Declaration of Competing Interest

The authors declare that they have no known competing financial interests or personal relationships which have, or could be perceived to have, influenced the work reported in this article.

## Appendix Supplementary materials

Image, application 1
